# Important Contributions to Reducing Nitrogen Oxide Emissions from Internal Combustion Engines

**DOI:** 10.3390/ijerph18179075

**Published:** 2021-08-27

**Authors:** Daniela Laura Buruiana, Adrian Sachelarie, Claudiu Butnaru, Viorica Ghisman

**Affiliations:** 1 Interdisciplinary Research Centre in the Field of Eco-Nano Technology, Advance Materials CC-ITI, Faculty of Engineering, “Dunarea de Jos” University of Galati, 47 Domneasca, 800008 Galati, Romania; daniela.buruiana@ugal.ro; 2 Faculty of Mechanical Engineering, “Gheorghe Asachi” Technical University of Iasi, 61–63 Mangeron Blvd, 700050 Iasi, Romania; butnaru.claudiu@yahoo.com

**Keywords:** emissions, risks, combustion dioxide

## Abstract

Nitrogen oxides are considered significant pollutants because of their effects on ecosystems and human health. The amount of NO_x_ emitted by internal combustion engines can be reduced, mostly by acting on the conditions in which combustion takes place, respectively by lowering the peak flame temperature, reducing the excess of oxygen, etc. The homogeneous charge compression ignition (HCCI) engine represents a new technology that can simultaneously reduce NO_x_ emissions and fuel consumption. This article presents these benefits of the HCCI engine by comparing the emissions and fuel consumption of a monocylinder engine when it is operated in a conventional way, with spark ignition, with those obtained when the engine is running in the HCCI mode. Moreover, since engine simulation has become an important tool for investigating the HCCI process and for developing new control strategies for it, this was used in this study to determine the effects of the exhaust gas recirculation on the combustion quality, respectively, on emissions.

## 1. Introduction

Air pollution is considered now the world’s largest environmental health threat, accounting for 7 million deaths around the world every year [1]. Air pollution causes and exacerbates a number of diseases, ranging from asthma to cancer, pulmonary illnesses, and heart disease. Outdoor air pollution and particulate matter, one of its major components, have been classified as carcinogenic to humans by the International Agency for Research on Cancer (World Health Organization). In accordance with recent estimates by the World Health Organization, exposure to air pollution is thus a more important risk factor for major non-communicable diseases than previously thought. Air pollution is the largest contributor to the burden of disease from the environment. The main substances affecting health are nitrogen oxides (NO_x_), Sulphur oxides (SO_x_), ozone, and particulate matter with the latter—especially particulate matter below 2.5 microns (PM_2.5_)—being of greatest concern, as these tiny particles penetrate deep into the lungs, affecting both the respiratory and vascular systems. Both extent and duration of the exposure influence health outcomes.

Nitrogen oxides are considered significant pollutants because of their effects on ecosystems and human health. Health experts have concluded that nitrogen oxides affect human health by causing lung damage, respiratory problems, and cancer [2,3,4]. To improve human health affected by NO_x_ pollution, research has shown that magnesium is an essential element in the human body, studies on the correlations between magnesium, depression, and toxicity in this context [5]. The NO_x_ is released into the atmosphere during combustion that takes place in heating equipment (furnaces, boilers, etc.) and in thermal machines (gas turbines, engines, etc.). In areas of high motor vehicle traffic, such as large cities, the amount of NO_x_ emitted into the atmosphere as air pollution can be significant, such as in Europe 40% of NO_x_ is emitted by road transport in accordance with reports from the European Commission and EU countries [6].

The amount of NO_x_ formed during combustion can be reduced, mostly, by acting on the conditions in which combustion takes place, respectively, by lowering the peak flame temperature, reducing the excess oxygen, etc. [7,8]. Recent studies show that NO_x_ emissions affect human health (especially for people with magnesium deficiency) such as cardiovascular disease, lung and respiratory dysfunctions, and neurology, as researchers from the medical domain search for correlations between diseases and the environment [9,10].

Nitrogen oxides are considered to have a significant impact on the environment: reacts with ammonia, moisture, and other compounds to form nitric acid vapor and related particles, which affects the ecosystems; reacts with volatile organic compounds in the presence of sunlight to form ozone; destroys ozone in the stratosphere; promotes lung diseases.

Diesel engines emit less CO_2_, but they emit significantly more NO_x_ (NO + NO_2_). Road transport contributes approximately 40% of the land-based NO_x_ emissions in the EU28 + countries (EU28 + Switzerland and Norway). The emissions from diesel cars have been attributed to a large part from the exceedance of ambient NO_2_ air quality values.

Recent studies are focused on the achievement of reductions in internal combustion (IC) engine CO_2_ and pollutant emissions (i.e., CO, HC, NO_x_, and particulates) [11,12,13].

In case of exposure to a high degree of pollution, there is a health risk and various diseases can occur. Regardless of the time of exposure to pollution, the results are negative for health. There are many sources of air pollution but one of the most important is that of cars. This type of pollution results from inefficient fuel combustion. Thus, Homogeneous Charge Compression Ignition (HCCI) combustion can be suitable for engine applications in order to obtain a maximum load. In large cities with heavy car traffic, the amount of nitrogen oxides emitted into the atmosphere is high and pollution becomes significant and indirectly becomes a health risk. Thus appear a series of respiratory diseases with inflammation of the airways at high levels. If the exposure is long-lasting, lung function may decrease and the response to allergens may increase [14,15].

The HCCI engine represents a new type of engine that combines the high performance of the compression ignition engine with the very low emissions of the spark ignition engine equipped with the three-way catalyst [16].

In an IC engine, HCCI combustion can be achieved by premixing the air-fuel mixture (either in the manifold or by early Direct Injection (DI)—such as in a spark ignition-SI engine) and compressing it until the temperature is high enough for autoignition to occur (such as in a Compressed Ignition (CI) engine). The HCCI burning is a method radically different from conventional spark ignition and compression-ignition burning. The combination of a diluted and premixed fuel-air mixture, with multiples areas of ignition in the combustion chamber, eliminates the areas with high combustion temperatures and prevents soot formation, thereby achieving very low NO_x_ and particulates emissions [17].

Using a lean, or more often diluted air-fuel mixture with exhaust gas recirculation (EGR) allows throttled operation of the gasoline HCCI engine, resulting in greater engine efficiency and lower fuel consumption than the spark ignition combustion. Therefore, the HCCI combustion is a technology that can simultaneously reduce the NO_x_ and particulate emissions of a diesel engine and has the capacity to achieve a reduction in fuel consumption and NO_x_ emissions of a gasoline engine [18]. Since HCCI engines operate with lean mixtures, the peak combustion temperatures are lower than those of a spark ignition engine and of a compression ignition engine. The lower peak temperatures prevent NO_x_ formation. However, low temperatures lead to incomplete combustion, especially near the chamber walls.

This leads to high carbon monoxide and hydrocarbons emissions. These emissions are not so important since they can be effectively removed by an oxidation catalyst because the exhaust gases are still rich in oxygen [19].

This study aims to reduce the fuel consumption and the NO_x_ emissions of an engine operating in HCCI mode compared to the conventional spark ignition operation. To achieve HCCI combustion and to control the burning process exhaust gas recirculation was used.

In addition, since numerical simulation has become an important tool for investigating the burning process and for developing new control strategies, due to its greater variability and lower costs compared to engines experiments, this tool was used in the current study.

The necessity of this research study is to comply with the stringent international regulations in order to develop advanced combustion strategies and the most significant reason is to diminuate the health impacts by reduction of NO_x_ emissions.

## 2. Materials and Methods

**Figure 1:**
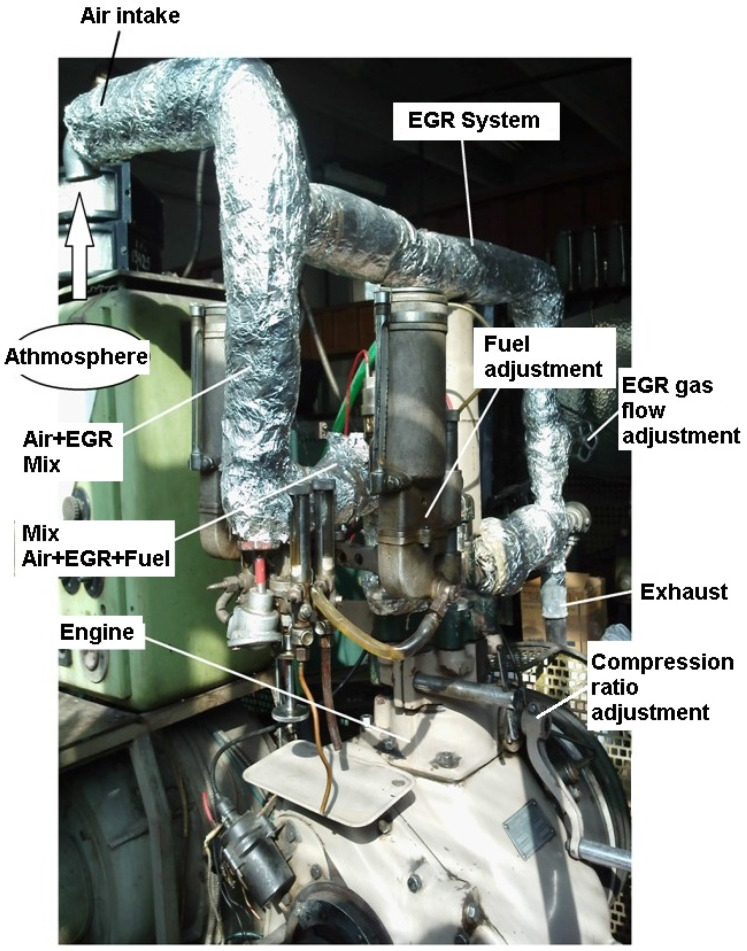
Experimental setup of the engine.

The experiments were carried out on the four-stroke monocylinder engine of the IT 9-2 experimental installation [20] using gasoline as fuel. The experimental engine is shown in Figure 1.

The engine characteristics are listed in Table 1. The fuel supply to the engine is achieved by a carburetor [21].

To obtain a more homogeneous mixture, first, we mixed the fresh air with the exhaust gases and after that, the resulted composition was mixed with gasoline.

For demonstrating the HCCI engine capacity to reduce nitrogen oxides we determined the emissions produced by the engine when it was operated as a conventional spark ignition engine and when it was operated in the HCCI mode.

### 2.1. Engine Parameters in Spark Ignition Operation Mode

The spark ignition engine relies on the spark given by the spark plug for igniting the air-fuel mixture resulting in a single flame front that spreads into the combustion chamber, with a distinct burned area and with the presence of the unburned areas [16,19]. While the flame spreads inside the cylinder, the mixture that ignites earlier is compressed at higher temperatures while the cylinder pressure continues to rise.

**Figure 2:**
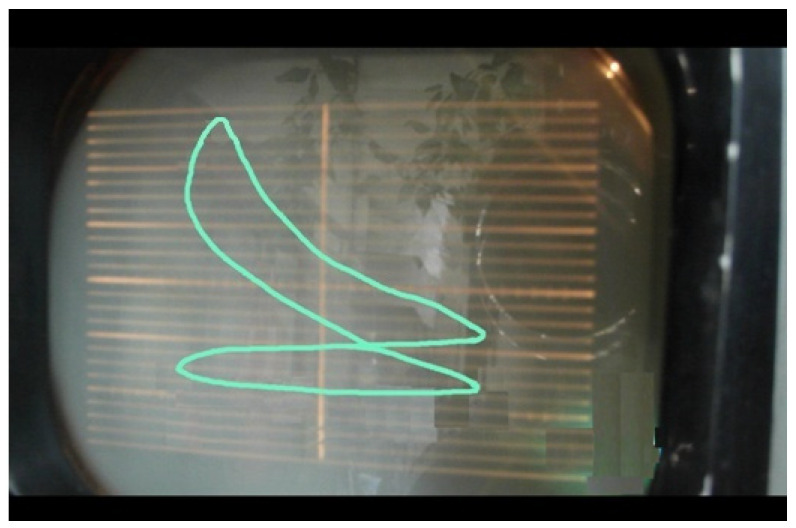
Indicated PV diagram for spark ignition operation mode.

During the experiments, we tried to determine the optimal combustion process by varying the fuel consumption and the spark timing. The effects of these parameters on the combustion process were monitored on the indicated diagram. After several attempts, we concluded that the optimum combustion process occurs when the spark time is set to 20 CAD before the top dead center and with a fuel consumption of 1.2 L/h. The resulted indicated diagram with cycle descriptions is shown in Figure 2.

Because the engine draws air directly from the atmosphere and is operated at a constant speed, the intake air flow is constant. After measuring the quantity of intake air resulted in an air flow of 12.9 m^3^/h.

Knowing that for burning one kilogram of gasoline we need an amount of 14.7 kg of air, we can calculate the excess air coefficient. In order to determine the excess air coefficient, we had to transform the flow rates in kg/h using Equation (1):

m = ρ × V(1)

where: m—mass; ρ– density; V—volume.

Knowing that the density of air is 1.1893 kg/m^3^ and the gasoline density is 0.73 kg/L results:

m_air_ = 1.1839 × 12.9 → m_air_ = 15.271 kg/h (2)

m_fuel_ = 0.73 × 1.2 → m_fuel_ = 0.876 kg/h (3)

The excess air coefficient is calculated using Equation (3):

(4) $$\lambda =\frac{AFR}{AFR_{stoich}}=\frac{\frac{15.272}{0.876}}{\frac{14.7}{1}}\to \lambda =1.18$$

Equation (3) results that the engine operates with a lean mixture.

After measuring the exhaust emissions of the spark ignition engine using gas analyzer SafeCheck 100, developed by Quest Technologies, resulted in a quantity of 52 ppm NO_2_ and 480 ppm CO.

### 2.2. Engine Parameters in HCCI Operation Mode

To achieve HCCI combustion in the experimental engine first we had to obtain the auto-ignition of the intake load. Auto-ignition represents the process by which the fuel begins to burn independently, without an external trigger such as the spark plug [22].

Like in the spark ignition engine, the air and the fuel in the HCCI engine are mixed in the intake system. The resulted mixture is then compressed. The auto-ignition of the mixture is initiated towards the end of the compression stroke, similar to the conventional compression ignition engine [23]. The charge temperature at the beginning of the compression stroke must be increased to achieve auto-ignition conditions at the end of the compression stroke. This is achieved by using exhaust gas recirculation [24]. This control strategy leads to a higher gas temperature along the compression stroke, which in turn speeds up the chemical reactions leading to the start of the burning of the homogeneous mixed air-fuel charge [25].

HCCI combustion is achieved by controlling the temperature, pressure, and the air-fuel mixture so that the auto-ignition process takes place at the right time without causing an early heat release [26]. As a consequence of the fact that there is not a direct control of the ignition timing, the initial conditions, as well as the internal flow will have a much greater impact on this type of combustion than for the conventional combustion process (spark ignition and compression ignition).

In order to achieve the HCCI combustion in the experimental engine, we tried to obtain a more homogeneous mixture by using a premixed chamber, and for increasing the load temperature and for controlling the combustion process inside the cylinder we used external exhaust gas recirculation [27]. In the experiments presented in this paper, we tried to achieve a maximum EGR degree in order to reduce the NO_x_ emissions and the fuel consumption.

Since the HCCI engine uses the heat of the previous cycle to achieve the combustion process, the engine was started in the conventional way as a spark ignition engine, and after reaching the operating conditions we made the transition to the HCCI mode. To achieve HCCI operation, we increased the intake load temperature by using EGR [28].

We tried to determine the optimal HCCI combustion process by varying the fuel consumption and the EGR ratio. After several tests, we established the necessary conditions for obtaining the HCCI combustion with the maximum EGR ratio (the EGR valve was fully opened):

− The initial air flow and fuel flow at the start of the engine in the spark ignition mode are those determined above: m_air_ =12.9 m^3^/h, m_fuel_ = 1.2 L/h;
− The EGR temperature is 220 °C;
− The temperature of the intake charge (air-EGR-fuel) is 180 °C;
− The intake air flow of air = 6.6 m^3^/h;
− The fuel consumption of fuel = 1.04 L/h.

**Figure 3:**
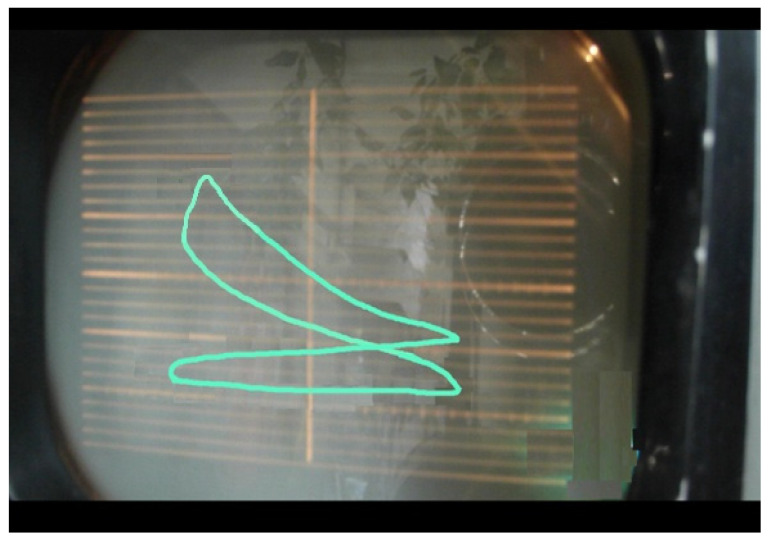
Indicated PV diagram for HCCI combustion mode.

The resulted indicated diagram that helped us to determine the optimum HCCI combustion process is shown in Figure 3.

After measuring the exhaust emissions obtained in the HCCI mode operation resulted in a quantity of 21 ppm NO_2_ and 1050 ppm CO.

As can be seen from the PV diagrams for spark ignition operation mode (Figure 2) and for HCCI combustion mode (Figure 3), the optimal parameters obtained are close to the ideal diagram of the Otto Cycle (PV diagram).

### 2.3. Numerical Simulation of the HCCI Engine

Because the HCCI combustion process is controlled by chemical kinetics, this type of combustion can be studied very well using numerical simulation. The development of accurate models to study the effects of different operating parameters and engine performance represents a great interest for experimental research. Analyzing these factors exclusively in the laboratory is expensive, inefficient, and impractical because many variables (e.g., the temperature inside the cylinder) are either difficult to measure or impossible to isolate due to complex interactions with other variables.

As a simulation program, we chose CHEMKIN-PRO, a program developed by Reaction Design (www.reactiondesign.com, accessed on 30 January 2021).

Since real fuels, in this case, gasoline, consist of several hundreds of compounds, the composition is too complex to be simulated. In order to simulate the gasoline characteristics, we used a gasoline surrogate consisting of 17% n-heptane (C_7_H_16_), 63% iso-octane (C_8_H_18_), and 20% toluene (C_6_H_5_CH_3_) [22]. The selected chemical kinetics mechanism to simulate the combustion process of the gasoline surrogate is developed by “Lawrence Livermore National Laboratory” (www-pls.llnl.gov, accessed on 30 January 2021.).

To simulate the HCCI operation mode of the experimental engine we used, in addition to the engine specifications given in Table 1, the engine specifications from Table 2 and the internal combustion engine model of the CHEMKIN-PRO program [25].

After processing the mechanisms data, we introduced the engine input parameters. It can be noted that the ambient temperature at which we performed the experiments was 300 K.

Table 3 lists all input data that were entered for simulating the HCCI engine.

To determine the influence of the EGR ratio on combustion and emissions we simulated three EGR ratios (10%, 30%, and 50%, respectively). In this case, we kept constant the fuel consumption (1.04 L/h) determined experimentally. Knowing that the intake airflow (without EGR) is 12.9 m^3^/h, we can calculate the intake airflow and the exhaust gas mass flow when using EGR with the following relationship:

(5) $$\overset{´}{m}=m*\left(1-\mathrm{EGR}\right)$$

where $\overset{´}{m}$: mass flow with EGR; *m*: mass flow without EGR.

The input parameters that change at the same time with varying the EGR ratio are given in Table 4.

## 3. Results and Discussion

As expected, we managed to achieve the HCCI combustion process with EGR in the experimental engine. From the experiments conducted, the following conclusions can be drawn.

The HCCI operating range has three main limiting factors: the misfire factor, in this area the concentrations are high enough to late the ignition and to increase the burning time; partial burning factor, in this area the combustion temperature is not high enough to allow complete combustion of the lean air-fuel mixtures; knock occurrence factor, in this area the heat release rates are high enough to cause pressure oscillations in the cylinder, phenomena observed from other authors [29].

The temperature of the mixture inside the cylinder and the EGR ratio are important factors that affect the combustion characteristics and the auto-ignition timing. The pressure inside the cylinder when the engine is operated in the HCCI mode is smaller than when the engine is operated in the spark ignition mode. In the HCCI engine, the fuel consumption and exhaust emissions can be reduced compared to conventional engines, because of the fact that many activities developed by human activities such as industry, producing energy [30,31,32].

Because when the engine was operated in the spark ignition mode, we achieved a fuel consumption of 1.2 L/h and when it was operated in the HCCI mode, we obtained a fuel consumption of 1.04 L/h, we can calculate the fuel economy achieved by the HCCI process as follows:

(6) $$\eta_{fuel}=\frac{m_{fuel}-{\overset{´}{m}}_{fuel}}{m_{fuel}}*100$$

(7) $$\eta_{fuel}=\frac{1.2-1.04}{1.2}*100\Rightarrow \eta_{fuel}=13.3\%$$

The NO_2_ reduction ratio when the engine is operated in HCCI mode compared to spark ignition operation mode can be calculated using:

(8) $${\mathrm{NO}}_{2}=\frac{{\mathrm{NO}}_{2\;SI}-{\mathrm{NO}}_{2\;HCCI}}{{\mathrm{NO}}_{2\;SI}}*100$$

(9) $${\mathrm{NO}}_{2}=\frac{52-21}{52}*100\;\Rightarrow \;{\mathrm{NO}}_{2}=60\%$$

Since the engine was operated at a constant speed, temperature, pressure, and the intake air flow when the engine was running without and with EGR are known, we can calculate the EGR ratio using the following equation:

(10) $$\mathrm{EGR}=\frac{m_{air}-{\overset{´}{m}}_{air}}{m_{air}}$$

(11) $$\mathrm{EGR}=\frac{12.9-6.6}{12.9}*100\Rightarrow \mathrm{EGR}=49\%$$

The high CO emissions obtained in the combustion mode are assumed to be mainly caused by the incomplete combustion in the limiting area, which is located near the combustion chamber wall [33]. There CO emissions are formed because of the flame extinction, which causes a lower temperature. Because of the lower temperature, the time necessary to complete the combustion process increases, and therefore, there is not enough time to complete the last stage of oxidation of the CO into CO_2_ [19].

The results obtained from the experimental tests showed that we achieved, in the HCCI operation mode compared to spark ignition operation mode, a fuel economy of 13.3% and a reduction in NO_2_ emissions of approximately 60% at an EGR ratio of 49%.

**Figure 4:**
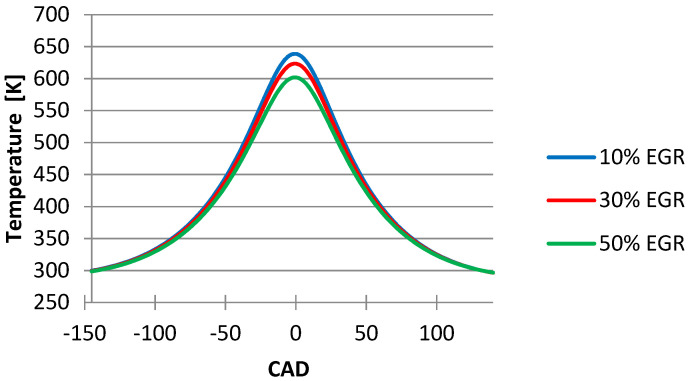
EGR ratio influence on temperature inside the cylinder.

**Figure 5:**
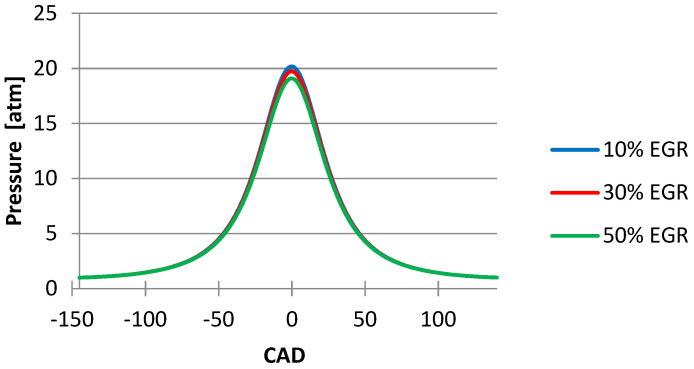
EGR ratio influence on pressure inside the cylinder.

Figure 4 shows the influence of the EGR ratio on the temperature inside the cylinder and it can be seen that when there are increases in the EGR ratio at 50% the temperature decreases. The same phenomena are observed in the graphic with the influence of the EGR ratio on the pressure inside the cylinder (Figure 5).

**Figure 6:**
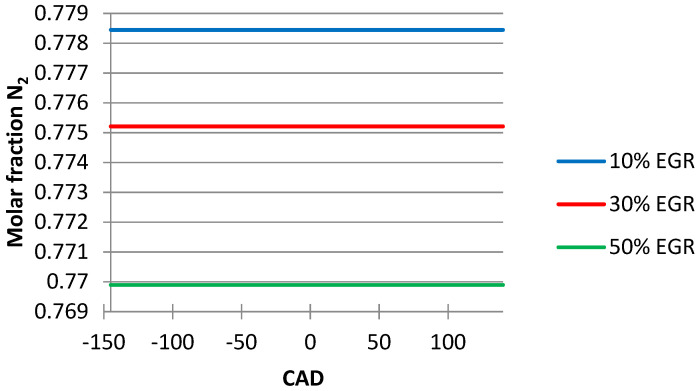
EGR ratio influence on N_2_ emissions.

In Figure 6, it can be seen that the EGR ratio influences N_2_ emissions, and the values obtained of the N_2_ emissions decrease with the increase in the EGR ratio at 50%.

**Figure 7:**
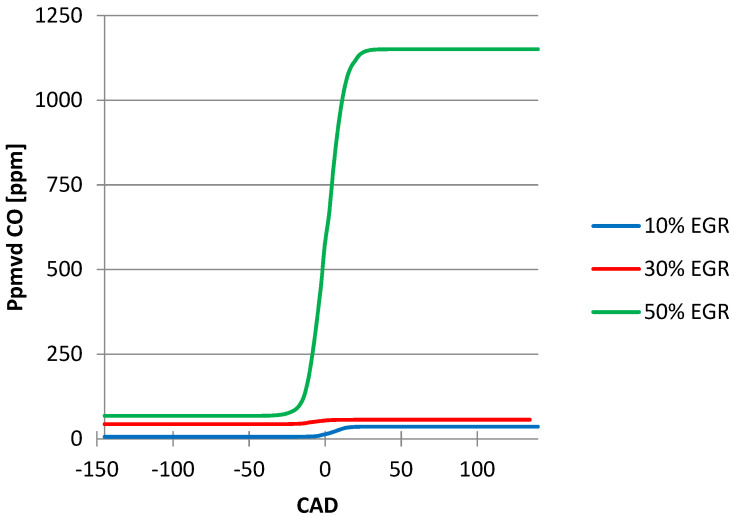
EGR ratio influence on CO emissions.

In the case of CO emissions with the EGR ratio (Figure 7), the curves for 10% and 30% EGR are similar, but when increased at 50% the shape of the curve is different in the positive domain with an increase in the CO emissions value.

**Figure 8:**
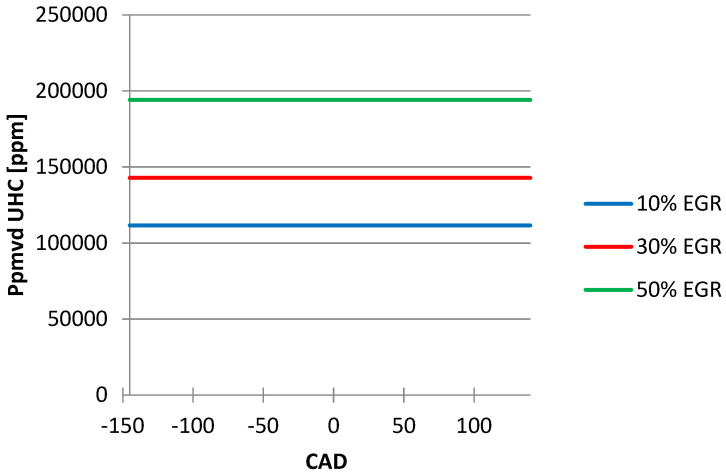
EGR ratio influence on UHC emissions.

Figure 8 shows that the values of UHC emissions increase with an increase in the EGR ratio.

From the graphics below (Figure 4, Figure 5, Figure 6, Figure 7 and Figure 8), obtained after simulating the HCCI engine, we noticed that the temperature and the pressure inside the cylinder decreased simultaneously with the increase in the EGR ratio, the most noticeable changes being obtained when we increased the EGR ratio from 30% to 50%. One explanation for this variation could be the fact that the excess air coefficient is not constant. The same happened with the N_2_ emissions.

We cannot say the same thing about the unburned hydrocarbon (UHC) emissions, their quantity increased once with the increasing EGR ratio because the amount of fresh air decreased while the fuel flow remained constant.

For the CO emissions, we noticed that they increased once with the increase in the EGR ratio, a significant increase occurred when we varied the EGR ratio from 30% to 50%.

After comparing the CO emissions obtained with the help of the simulation program (1150 ppm) with those obtained in the experimental tests (1050 ppm) when we used the same input parameters, we can conclude that the simulation program is a good tool for determining the HCCI combustion characteristics.

One explanation for the obtained difference is that in the simulation program we used a gasoline surrogate because gasoline has a too complex composition in order to be simulated.

The methodological novelty of this research is building the experimental engine to optimize the HCCI phenomenon in order to combat the negative health effects on the population at large. With this engine, we can modify/adjust: the compression ratio; EGR gas flow; fine level of fuel adjustment; fresh air and EGR gases mixture adjustment; fresh air, EGR gases mixture, and fuel adjustment.

## 4. Conclusions

Nitrogen oxides are one of the key pollutants that produce serious health issues, and it is well known at the Mondial level the exposure to ambient nitrogen dioxide has been linked to increased mortality.

The motivation of our study is to diminuate health risk by a significant reduction in the NO_x_ emissions from the internal combustion engine, especially in Romania where the average age of vehicles is higher than 15 years—affirmed by the Committee of Inquiry into Emission Measurements Automotive Sector (EMIS).

HCCI combustion is achieved by controlling the temperature, pressure, and air-fuel mixture so that self-ignition takes place at the appropriate time without any earlier heat release. As a consequence of the fact that there is no direct control over the ignition moment, the initial conditions, as well as the internal flow, will have a much greater impact on this type of combustion than conventional combustion processes (with spark ignition and compression-ignition).

To obtain the HCCI combustion in the experimental engine, we tried to obtain a mixture as homogeneous as possible by using a premix chamber, and to increase the temperature of the mixture and to control the combustion inside the cylinder we used the external recirculation of exhaust gases.

In this paper, we have tried to achieve the highest possible degree of recirculation in order to reduce emissions and fuel consumption.

In order to obtain the HCCI operation, we increased the load intake temperature by using the external recirculation of the exhaust gases.

It is clear that the HCCI technology provides superior efficiency regarding fuel consumption and NO_x_ emissions compared to conventional engines with spark ignition. It is not yet so certain the ability of these engines to deliver these features cheap and, perhaps more importantly, to be reliable through the entire life of the vehicle. Continuous progress in electronic control brought the HCCI concept to the stage of functional reality, but it still needs some adjustment in order to achieve vehicles production.

## Figures and Tables

**Table 1:** Experimental engine characteristics.

Type	Four Stroke, One Cylinder
Cylinder diameter	85 mm
Piston stroke	115 mm
Connecting rod length	300 mm
Cylinder displacement volume	0.652 L
Compression ratio	10
Speed	Constant, 900 rpm
Number of valves per cylinder	2
Fuel	Gasoline (RON 95)
Ignition	Spark ignition
Air supply	Naturally aspirated
Cooling	with water
Start	With a 7 KW electric engine

**Table 2:** Experimental engine characteristics.

Intake Valve Opening Time	10 CAD ATDC
Intake valve closing time	215 CAD ATDC
Exhaust valve opening time	500 CAD ATDC
Exhaust valve closing time	10 CAD ATDC

**Table 3:** Input data for simulating the HCCI engine.

	Parameter	Value	Measurement Unit	Observations
Intake air	Temperature	300	K	
	Flow rate	3.818	g/s	Varies according to the EGR ratio.
	Composition	O_2_ = 21	%	
		N_2_ = 79		
Fuel	Temperature	300	K	
	Flow rate	0.211	g/s	
	Composition	C_7_H_16_ = 17	%	
		C_6_H_5_CH_3_ = 20		
		C_8_H_18_ = 63		
Air + EGR Reactor	Residence time	0.0055	s	Varies according to the speed.
	Temperature	460	K	
	Pressure	1	atm	
Air + EGR + Fuel Reactor	Residence time	0.0083	s	Varies according to the speed.
	Temperature	450	K	
	Pressure	1	atm	
HCCI engine	Simulation time	280	CAD	
	Compression ratio	10	-	
	Engine capacity	0.652	cm^3^	
	Connecting rod to crank radius ratio	2.608	-	
	Speed	900	rpm	
	Starting crank angle (ATDC)	−145	CAD	
	Coefficient a	0.035	-	
	Coefficient b	0.71	-	
	Coefficient c	0	-	
	Chamber bore diameter	8.5	cm	
	Prandtl Number	0.7	-	
	Coefficient c2	3.24	-	
	Coefficient c11	2.28	-	
	Coefficient c12	0.308	-	
Heat_Loss Reactor	Residence time	0.0426	s	Varies according to the speed.
	Temperature	500	K	
	Pressure	1	atm	
	Volume	1013.41	cm^3^	
	Internal surface area	922.73	cm^2^	
	Heat transfer coefficient	0.00009	cal/cm^2^·K·s	
	Ambient temperature	300	K	
	Exhaust gas flow rate	4.544	g/s	Varies according to the air and fuel flow and to the EGR ratio.
Splitter Reactor	The amount of EGR	10	%	The sum of these quantities must be equal with 1.
	The amount of exhaust gases	90	%	

**Table 4:** Input parameters of the HCCI engine influenced by the EGR ratio.

EGR Ratio [%]	Intake Air Flow Mass [g/s]	Exhaust Gas Flow Mass [g/s]
10	3.818	4.476
30	2.9696	4.544
50	2.1211	4.762
